# The association of fecal calprotectin and respiratory exacerbation in cystic fibrosis patients

**DOI:** 10.1186/s12876-022-02553-x

**Published:** 2022-11-28

**Authors:** Farid Imanzadeh, Fatemeh Kerami, Maryam Hassanzad, Amirhossein Hosseini, Mahmoud Hajipour, Ghamartaj Khanbabaee, Naghi Dara, Katayoun Khatami, Nazanin Farahbakhsh, Aliakbar Sayyari

**Affiliations:** 1 Pediatric Gastroenterology, Hepatology and Nutrition Research Center, Research Institute for Children’s Health, Shahid Beheshti University of Medical Sciences, Tehran, Iran; 2 Department of Pediatrics, School of Medicine, Shahid Beheshti University of Medical Sciences, Tehran, Iran; 3 Pediatric Respiratory Diseases Research Center, NRITLD, Masih Daneshvari Hospital, Shahid Beheshti University of Medical Sciences, Tehran, Iran; 4 Pediatric Pulmonology Department, Mofid Pediatric’s Hospital, Shahid Beheshti University of Medical Sciences, Tehran, Iran

**Keywords:** Fecal Calprotectin, Cystic fibrosis, Pulmonary exacerbations

## Abstract

**Background:**

CF patients experience several episodes of pulmonary exacerbations and reduction in their lung function progressively. Lung function is not the only diagnostic index by physicians to decide if CF patients require antibiotic therapy following pulmonary exacerbations. Non-invasive fecal indicators are increasingly being used to assess intestinal inflammation. Calprotectin is the most extensively utilized fecal biomarker in recent CF researches.

**Methods:**

In this longitudinal study, 30 CF patients (1–18 years) without current infectious gastroenteritis were recruited from Mofid Children's Hospital and Masih Daneshvari Hospital, Tehran, Iran. Then, fecal calprotectin levels were evaluated before treatment, two weeks after systemic antibiotic administration, as well as recurrence of pulmonary exacerbation after first post-hospital discharge.

**Results:**

The initial fecal calprotectin level in CF patients receiving antibiotics was 651.13 ± 671.04, significantly decreasing two weeks after antibiotic therapy and following recurrence (171.81 ± 224.40, 607.93 ± 549.89, respectively; *P* < 0.01). Following systemic antibiotic treatment, the patient's respiratory and GI symptoms improved (*P* < 0.01).

**Conclusion:**

Our findings revealed that fecal calprotectin modifications are associated with CF pulmonary exacerbations and antibiotic treatment could reduce calprotectin levels. Therefore, the fecal calprotectin level could be considered as a diagnostic tool and an index to follow the response to treatment in CF pulmonary exacerbations.

## Introduction

Cystic fibrosis (CF) is a life-threatening autosomal recessive disease and CF patients experience several episodes of pulmonary exacerbations and reduction in their lung function progressively [1]. CF is a multisystem disease with a high risk of GI irritation. The gut and the respiratory tract endure a typical triad of mucus stasis, infection, and inflammation [2].

An acute pulmonary exacerbation includes flaring up or worsening of the signs and symptoms such as cough, excessive mucus, and dyspnea [3]. Importance of respiratory exacerbations is directly related to the acute decrease of lung function which is often not recoverable by antibiotic therapy [1].

The major causes of morbidity and mortality in CF patients are bronchiectasis, small airway blockage, and progressive respiratory dysfunction, which leads to chronic and recurrent exacerbations [4]. Furthermore, respiratory exacerbations and decreased life expectancy of patients CF are closely associated [1].

Gastrointestinal (GI) symptoms, such as malabsorption, steatorrhea, obstruction, and insufficient weight gain are among the first symptoms of CF [5, 6]. Moreover, elevated inflammatory blood markers have been found in CF patients, which is correlated with disease severity [7–9].

Lung function is not the only diagnostic index by physicians to decide if CF patients require antibiotic therapy following pulmonary exacerbation. Non-invasive fecal indicators are increasingly being used to assess intestinal inflammation. Calprotectin is the most extensively utilized fecal biomarker in recent CF researches [10]. Calprotectin is a zinc- and calcium-binding protein produced mainly by neutrophils. Besides, minor levels of calprotectin have been detected in monocytes and reactive macrophages. This compound has bacteriostatic and fungistatic properties, with minimal inhibitory doses comparable to antibiotics [11, 12]. It has a regulatory role in inflammatory processes which mainly derived from neutrophils and eosinophils [2].

Patients with pancreatic insufficiency have presented higher levels of fecal calprotectin, which may be linked to CF findings [7]. Additionally, several studies have confirmed that sputum and serum calprotectin levels decrease during and after antibiotic therapy in acute respiratory exacerbations in CF patients [2]. Moreover, it has been claimed that the fecal calprotectin level could be used to assess the effect of new medications on the intestine of CF patients [13].

We have designed this research to study the association between fecal calprotectin as a predicting index for respiratory exacerbation in CF patients with the main goal of improving the paradigm of CF diagnosis and treatment.

## Material and methods

This longitudinal study recruited all CF children Between 1 to 18 years old with respiratory exacerbation, collected from Mofid Children's Hospital and Masih Daneshvari Hospital, Tehran, Iran, between 2018 and 2020. The Cystic Fibrosis Foundation diagnosis criteria were met by all CF children and the available subjects were included by sequential sampling [14]. Patients were excluded from the study if they had acute exudative diarrhea or chronic diarrhea. Patients with a known pre-existing GI bleeding at the time of sample collection were excluded. we should note that all CF patients had pancreatic insufficiency based on laboratory data of (fecal elastase and trypsinogen enzyme activity); all of whom were under enzyme replacement therapy.

The study was approved by the relevant local ethics committee (IR.SBMU.MSP.REC.1398.867). Informed consent was obtained from patients or their parents.

Thirty cases with CF entered our study. Demographic data (age, sex, weight, height, BMI z-score and weight for length z-score) were noted from hospital recordings. Additionally, clinical features (cough, hemoptysis, dyspnea, increased sputum, sputum color change, fever, weight loss, pallor, osteoporosis, anemia, weakness and lethargy, clubbing, tooth decay, weight loss or failure to weight gain and respiratory diarrhea) were recorded in pre-prepared questionnaire.

Since CF is characterized by recurrent flare-ups of respiratory symptoms and based on the previous researches, CF individuals ' fecal samples were collected at the first visit, two weeks after antibiotic therapy, and after first recurrence of CF exacerbation [15, 16]. Calprotectin levels were measured before and after antibiotic therapy ( Amikacin + Azithromycin) in CF patients who did not have gastroenteritis, chronic diarrhea or gastrointestinal bleeding. Patients were followed for six months, and disease recurrence was analyzed to link calprotectin levels and disease recurrence.

The samples were kept at -20 °C until they were transferred to the laboratory and then they were kept at -80 °C until analysis. The PhiCal kit (Calpro, San Diego, CA, US) was used to extract and measure calprotectin from frozen fecal samples according to the manufacturer's instructions. The upper normal limit of the kit was < 50 µgr/gr according to the manufacturer.

Statistical analysis was performed using SPSS Statistics 23.0 (Illinois, USA). Quantitative variables were presented as mean ± SD. Normal distributions of variables were performed using Kolmogorov–Smirnov test. Paired t-test was used to compare quantitative variables for two time-points if the variables showed normal distribution and Wilcoxon test was used for non-parametric distributions. If the variables were normally distributed, the repeated measure ANOVA was used to compare them at different time points, and if they were not, the Kruskal–Wallis test was used. Kaplan Meyer diagram was used to examine the relapse. *P* < 0.05 was considered statistically significant for all analyses.

## Results

A total of 30 CF patients with respiratory exacerbation were collected, including 14 (46.7%) females and 16 (53.3%) males. 16.7% were under 6, 36.7% were 7–12 and 46.7% were 13–18 years old. The BMI z-score of patients over the age of two was calculated. Patients under the age of two were assessed using a z-score based on their weight/height index [15].

The mean age of CF patients was 11.53 ± 5.43 years old. Out of 30 patients with CF disease, 28 had BMI Z-score > -2, with a minimum of -3.5 and a maximum of 1. The demographic features of the patients are detailed in Table 1.

**Table 1:** Demographic characteristics of CF patients

**Variable**	**Number**	**Mean**	**Standard Deviation**	**Minimum**	Maximum
Age	30	11.53	5.43	1	18
Weight	30	30.21	14.06	4	58
Height	30	132.11	29.53	50	170
Z-score BMI (≥ 2-year-old)	28	-1.31	1.31	-3.5	1

The clinical symptoms of patients with CF were evaluated at the first visit, two weeks later, and by the next pulmonary exacerbation. As shown in Table 2, the most prevalent clinical symptoms at the first visit were cough (80%), increased sputum (60%), and shortness of breath (50%). Two weeks later, cough (36.7%), increased sputum (20%), and sputum discoloration (20%) were the most prevalent clinical signs. After a recurrence of disease, cough, increased sputum, and shortness of breath were the most common symptoms, accounting for 90%, 56.7%, and 46.7%, respectively.

**Table 2:** Clinical features of CF patients

Clinical manifestation	Frequency (percentage) of the first visit	Frequency (percentage) in two weeks later	Frequency (percentage) of relapse with symptoms
**Cough**	(80) 24	(36.7) 11	(90.0) 27
**Hemoptysis (severe (> 250 ml/day)**	(6.7) 2	(6.7) 2	(16.7) 5
**Dyspnea**	(50) 15	(13.3) 4	(46.7) 14
**Increased sputum**	(60) 18	(20) 6	(56.7) 17
**Change the color of sputum**	(26.7) 8	(20) 6	(16.7) 5
**Fever**	(6.7) 2	(6.7) 2	_
**Weight loss**	(20) 6	(6.7) 2	(3.3) 1
**Anemia**	(3.3) 1	_	_
**Weakness and lethargy**	(10) 3	(3.3) 1	(3.3) 1
**failure to weight gain / weight loss**	(20) 6	(6.7) 2	(10) 3
**Other manifestations**	(38.8) 12	_	_

**Fig. 1:**
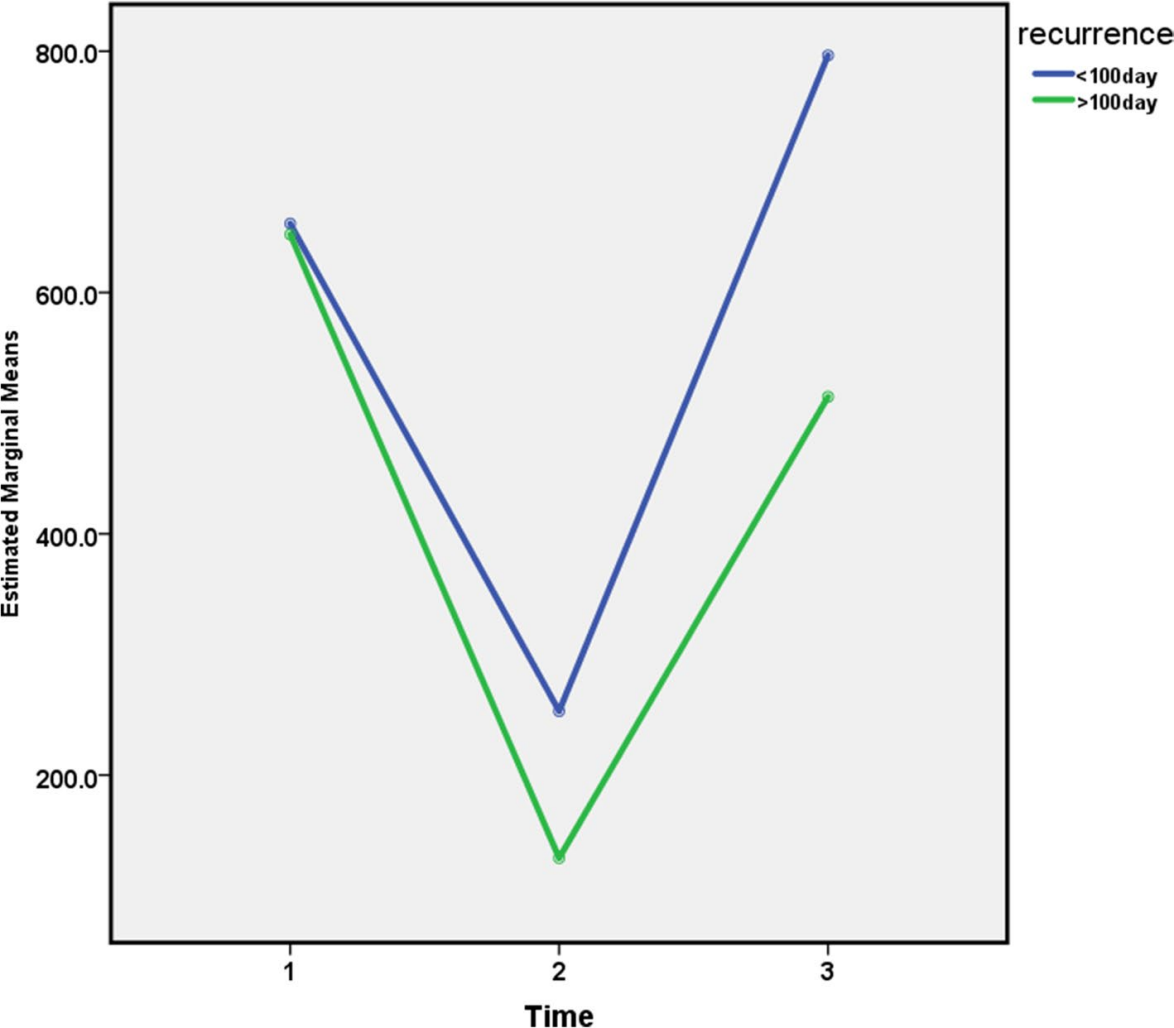
A schematic image of relapse in patients by the referral day(before and after 100 days)

After 100 days, there was a 50% rate of recurrence (15 patients) among the patients (Fig. 1). We observed a significant difference in levels of fecal calprotectin following antibiotic therapy. Calprotectin levels in patients with CF were 651.13 ± 671.04 μg/g at the initial visit, 171.81 ± 224.40 μg/g two weeks after antibiotic treatment, and 607.93 ± 549.89 μg/g after recurrence. According to these repeated assessments, the levels of calprotectin in patients have reduced after medical treatment of pulmonary exacerbation and increased on the first relapse during their follow-up. This association was statistically significant (*P* < 0.001) (Table 3).

**Table 3:** Descriptive Statistics of exacerbation relapse by the time of referral (before 100 days or after 100 days)

recurrence		N	Minimum	Maximum	Mean	Std. Deviation
< 100 day	cal1	10	123.7	2039.0	657.170	592.6269
	cal2	10	20.6	1233.0	252.950	373.5518
	cal3	10	203.0	2437.0	796.600	721.3737
	Valid N (listwise)	10				
> 100 day	cal1	20	61.2	2544.0	648.120	721.7451
	cal2	20	12.0	320.0	131.250	74.6119
	cal3	20	87.9	1400.0	513.595	432.3512
	Valid N (listwise)	20				

**Fig. 2:**
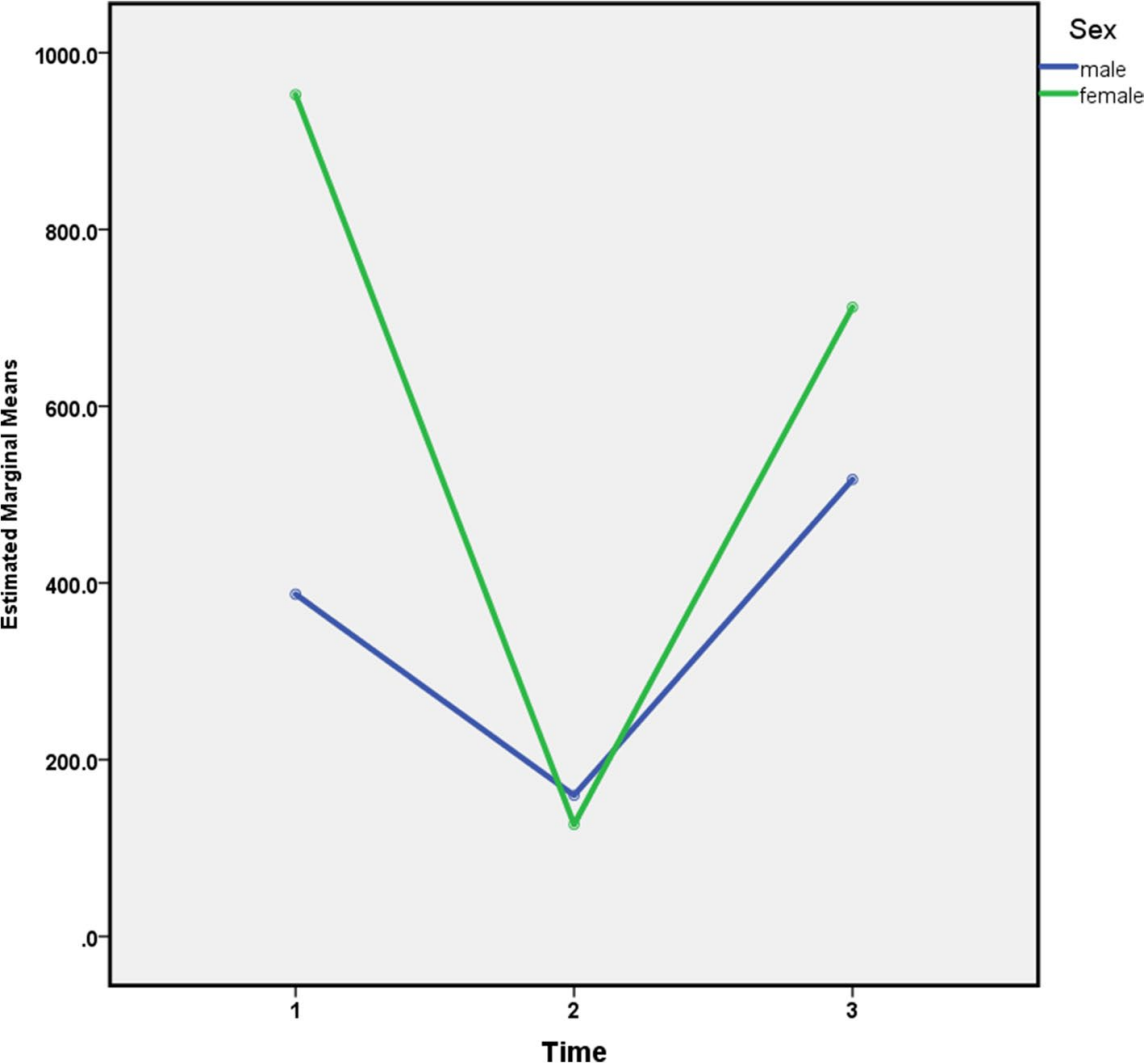
Fecal calprotectin level in different times by gender (1: At the time of exacerbation, 2: Two weeks after antibiotic therapy, 3: Relapse)

**Fig. 3:**
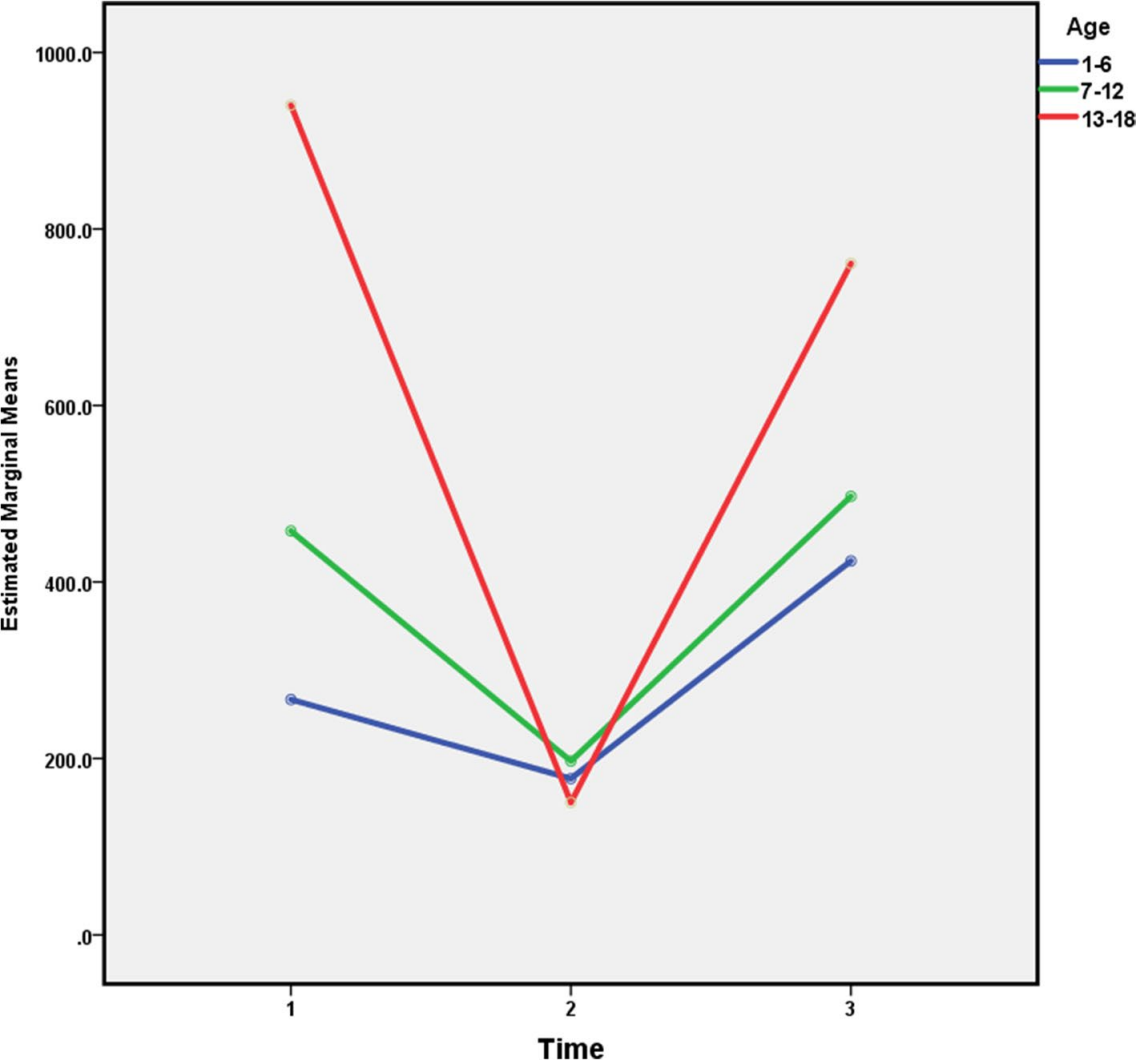
Fecal calprotectin level in different times by age (1: At the time of exacerbation, 2: Two weeks after antibiotic therapy, 3: Relapse)

According to the results of the analysis, there was a significant association between calprotectin levels and gender and between calprotectin and age at the first visit (*P* < 0.05). The mean calprotectin level in females admitted at the hospital with respiratory exacerbation was substantially greater than that of male patients at the first visit. However, calprotectin levels decreased in both females and males after antibiotic therapy and the antibiotics' effectiveness was relatively high in both groups (*P* < 0.05). There was no significant link between calprotectin level, gender, or age two weeks after treatment and after the recurrence of symptoms (*P* > 0.05) (Figs. 2 and 3).

At the time of the first visit, the mean level of calprotectin in the age group of 1–6 years was significantly lower than the age groups of 7–12 years and 13–18 years. Three groups responded to treatment after two weeks of treatment, with the mean level of calprotectin reaching 200 μg/g. Following a recurrence of disease, the age group of 13–18 years showed a more significant increase in the level of calprotectin than the age groups of 1–6 years and 7–12 years.

The average calprotectin level in the first visit in females with respiratory exacerbation was significantly higher in comparison to the males with respiratory exacerbation (900 μg/g *vs.* 400 μg/g). Calprotectin levels in both genders decreased following antibiotic therapy in the second visit, and their average was equal. After a recurrence of disease in females, calprotectin showed a more significant increase than in males.

To summarize; the calprotectin levels in patients have reduced after medical treatment of pulmonary exacerbation and have increased on the first relapse of exacerbation during their follow-up. Moreover, the average calprotectin level in the first visit in females with respiratory exacerbation was significantly higher. After a recurrence of the disease in females, calprotectin level showed a more significant increase than in males. Finally, following a recurrence of disease, the age group of 13–18 years showed a more significant increase in the level of calprotectin than the age groups of 1–6 years and 7–12 years. These associations were all statistically meaningful. Also, there was a significant association between calprotectin levels and gender and also between calprotectin and age at the first visit.

## Discussion

The current study reported an association between fecal calprotectin levels and respiratory exacerbation in CF patients referred to the Mofid Children's Hospital and Masih Daneshvari Hospital between 2018 and 2020. We observed that the fecal calprotectin level is affected at the initiation of the pulmonary exacerbation and also after initiation of antibiotic therapy.

Similarly, increased fecal calprotectin levels were detected in CF patients during a CF exacerbation [2]. Gray discovered that patients with a serum calprotectin concentration of < 9.1 μg/mL at the end of an exacerbation had a longer interval between exacerbations than those with calprotetin > 9.1 μg/mL [17].

Apart from the underlying genetic abnormality, continuous use of antibiotics and pancreatic enzymes, have altered bowel motility, and the gut microbiome have been linked to intestinal inflammation [18]. Treatment using systemic antibiotics for pulmonary exacerbations has been demonstrated to improve systemic inflammatory indicators and also serum and sputum calprotectin levels. Antibiotic therapy for pulmonary symptoms may alleviate the inflammatory condition of the GI tract. The prospective pilot study conducted by Schnapp et al.on 14 CF patients found that the influence of antibiotic treatment on the gut microbiome may lead to diminished fecal calprotectin levels [2].

In our study, fecal calprotectin levels were measured before and after 14 days of antibiotic therapy. A significant association was observed between antibiotic administration and fecal calprotectin levels. It was observed that the levels of calprotectin declined considerably(*P* < 0.001). Dong et al*.* revealed that a change in serum calprotectin levels during the first 28 days of azithromycin therapy predicted the risk of pulmonary exacerbation by day 168 [19]. It seems that antibiotic therapy changes the microbiome in the GI tract and the pulmonary system by decreasing the inflammatory burden [2].

The diagnostic performance of calprotectin cut-offs was investigated in a longitudinal research to distinguish between stable and pulmonary exacerbation visits. It has been found that calprotectin had optimal absolute and fold-change thresholds of 8.1 mg/L and 1.3-fold, respectively [20]. Our findings support previous research that calprotectin is a potential biomarker of pulmonary exacerbation risk in CF patients by demonstrating that fecal calprotectin is responsive to antibiotic therapy. More importantly, these changes in fecal calprotectin levels are associated with the risk of pulmonary exacerbation. Moreover, according to recent researches, serum calprotectin would be a predictive diagnostic biomarker of pulmonary exacerbation risk in CF patients and reduces the response to treatment [17, 20, 21].

The study of Jung D et al. claims that serum calprotectin besides serum CRP would be able to discriminate pulmonary exacerbation conditions from stable visits. They believe that designation a step-wise algorithm of cut-off points and fold-change of both above-mentioned markers would improve their diagnostic performance [22]. This is compatible with our results; however, the study by Grag et al. have concluded that fecal calprotectin levels in CF patients is completely age-dependent. Thus, a very precise interpretation of calprotectin is needed to be used in CF patients’ treatment of pulmonary exacerbations, particularly in patients less than 4 years old [11].

A study of Adriaanse et al. have demonstrated that there is an inverse correlation between intestinal inflammation and lung function of CF patients. In this study, no correlation was found between fecal and sputum calprotectin, while fecal calprotectin level was strongly correlated with the presence of diabetes and exocrine pancreatic insufficiency. They finally concluded that the significant associations of enteropathy and CF morbidities highlight the relevance, clinically. These results were in contrast with our results. They also have claimed that no relation was found between calprotectin levels in feces and sputum [18].

As an additional point, we discovered that following a recurrence of disease, the age group of 13–18 years showed a more significant increase in the level of calprotectin than the age groups of 1–6 years and 7–12 years. In the research of Garg et al. they have studied the age-dependent variation of fecal calprotectin in CF children compared to healthy children. Overall, they reported that fecal calprotectin levels were different in CF patients and healthy children from 0 to 10 years and there was an upward trajectory until 4 Years. From 4 to 10 years, calprotectin was higher in CF Children compared with healthy ones. They have finally concluded that fecal calprotectin levels in children were age-dependent and had distinct trajectories. They finally mentioned that a precise interpretation of calprotectin should be done in drug trials specially in children less than 4 years old [23].

Moreover, we found out that the average calprotectin level in the first visit in females with respiratory exacerbation was significantly higher. After a recurrence of the disease in females, calprotectin level showed a more significant increase than in males. Finally, these associations were all statistically meaningful. The cohort study by Bathe et al. have concluded that gender does not have a significant effect on fecal calprotectin levels [24]. However, the studies are limited on this topic and further researches is needed.

## Conclusion

As conclusion, we detected higher fecal calprotectin levels in CF patients at the onset of the pulmonary exacerbation which had influenced following antibiotic therapy. Alteration of fecal calprotectin levels in individuals with CF upon systemic antibiotic administration reveals that GI inflammation is a part of a systemic response during a pulmonary exacerbation, contributing to understanding the mechanism and therapeutic approaches. Reduced fecal calprotectin levels in response to completed systemic antibiotic therapy for respiratory exacerbation shows that inflammation in the GI tract and the respiratory system is affected, presumably through the role of the intestinal microbiome.

Our findings revealed that fecal calprotectin modifications are associated with CF pulmonary exacerbations and antibiotic treatment could reduce calprotectin levels. Therefore, the fecal calprotectin level could be considered as a diagnostic tool and an index to follow the response to treatment in CF pulmonary exacerbations.

CF exacerbation is a systemic inflammatory process which affects the calprotectin level in either stool and sputum, as well as the serum calprotectin level; hence, such differentiation is of secondary clinical importance. However, further researches in the future may be needed to distinguish the fecal and sputum calprotectin changes.

Regarding to the small sample size, lack of enough patients’ cooperation, future studies with larger sample size and other inflammatory biomarkers should be considered in order to detect the most reliable index with an acceptable positive predictive value for CF exacerbations. Evaluation of microbiome composition may also contribute to understanding the pathologies underlying GI tract problems in CF patients in relation with pulmonary exacerbations.

## Data Availability

The data generated by and used in the study is available from the corresponding author upon reasonable request.
